# Enhancing cotton whitefly (*Bemisia tabaci*) detection and counting with a cost-effective deep learning approach on the Raspberry Pi

**DOI:** 10.1186/s13007-024-01286-0

**Published:** 2024-10-20

**Authors:** Zhen Feng, Nan Wang, Ying Jin, Haijuan Cao, Xia Huang, Shuhan Wen, Mingquan Ding

**Affiliations:** The Key Laboratory for Quality Improvement of Agricultural Products of Zhejiang Province, College of Advanced Agricultural Sciences, Zhejiang A&F University, Linan, Hangzhou, 311300 Zhejiang China

**Keywords:** Whitefly counting, Monitoring, Deep learning, The Raspberry Pi, Pest distribution

## Abstract

**Background:**

The cotton whitefly (*Bemisia tabaci*) is a major global pest, causing significant crop damage through viral infestation and feeding. Traditional *B. tabaci* recognition relies on human eyes, which requires a large amount of work and high labor costs. The pests overlapping generations, high reproductive capacity, small size, and migratory behavior present challenges for the real-time monitoring and early warning systems. This study aims to develop an efficient, high-throughput automated system for detection of the cotton whiteflies. In this work, a novel tool for cotton whitefly fast identification and quantification was developed based on deep learning-based model. This approach enhances the effectiveness of *B. tabaci* control by facilitating earlier detection of its establishment in cotton, thereby allowing for a quicker implementation of management strategies.

**Results:**

We compiled a dataset of 1200 annotated images of whiteflies on cotton leaves, augmented using techniques like flipping and rotation. We modified the YOLO v8s model by replacing the C2f module with the Swin-Transformer and introducing a P2 structure in the Head, achieving a precision of 0.87, mAP_50_ of 0.92, and F1 score of 0.88 through ablation studies. Additionally, we employed SAHI for image preprocessing and integrated the whitefly detection algorithm on a Raspberry Pi, and developed a GUI-based visual interface. Our preliminary analysis revealed a higher density of whiteflies on cotton leaves in the afternoon and the middle-top, middle, and middle-down plant sections.

**Conclusion:**

Utilizing the enhanced YOLO v8s deep learning model, we have achieved precise detection and counting of whiteflies, enabling its application on hardware devices like the Raspberry Pi. This approach is highly suitable for research requiring accurate quantification of cotton whiteflies, including phenotypic analyses. Future work will focus on deploying such equipment in large fields to manage whitefly infestations.

## Introduction

Cotton is a global economic crop, serving as a major source of natural fiber as an essential oil and protein crop [1, 2]. However, cotton production faces significant threats from various unfavorable factors, including diseases and pests. Among these, the whitefly *Bemisia tabaci* (Gennadius) stands out as a global agricultural and horticultural pest that causes direct damage to plants through feeding and indirect damage through the transmission of plant viruses [3–5]. With a wide range of over 1000 host plants, *B. tabaci* demonstrates remarkable adaptability, primarily affecting crops such as cotton, soybeans, and vegetables. *B. tabaci* has increasingly posed a serious threat worldwide, resulting in substantial economic losses [6].

*Bemisia tabaci* presents a substantial threat due to its rapid distribution, broad host range, high reproductive capacity, and devastating impact. Coupled with extensive cotton cultivation areas, *B. tabaci*s distribution complicates chemical control efforts in cotton fields [7, 8]. Furthermore, *B. tabaci*s ability to produce 11 to 15 generations annually, with significant generational overlap, underscores the importance of effective management throughout the cotton growth cycle. Overall, *B. tabaci*s minuteness and distribution pose challenges for detection, leading to a shortage of effective monitoring and early warning techniques.

Integrated Pest Management (IPM) strategies necessitate regular field sampling to accurately count and identify whitefly life stages for informed decision-making [9]. Traditional methods, reliant on highly trained personnel, are labor-intensive, costly, and variable due to expertise differences. Therefore, efficient and precise techniques for whitefly identification and enumeration are essential [10]. Current management practices, such as trap cropping, pesticide use, and breeding resistant cotton varieties, though useful, are costly, time-consuming, and environmentally risky [11,14]. Early and accurate field detection of whiteflies can significantly reduce labor, costs, and infestation levels, enhancing control effectiveness [15, 16]. Thus, there is a critical need for a rapid, convenient method to identify *B. tabaci*.

In recent decades, deep learning has been extensively applied for crop pest identification. For instance, Shen et al. developed a Faster R-CNN-based method for recognizing six common adult stored-grain insects under varying illumination conditions using FFDB [17]. Amarathunga et al. introduced a novel approach employing the Vision Transformer (ViT) architecture for fine-grained classification of two microscopic insects: the Western Flower thrips and Plague thrips, utilizing deep learning [18]. Both studies apply deep learning to identify pests in common field crops. Sun et al. developed TCME-YOLOv5 to assess pest infestation levels and created an intelligent pest identification system [19]. Additionally, a rapid plant insect resistance testing instrument has been developed, incorporating an automated algorithm for quantifying whitefly eggs [20]. Li et al. have advanced precision agriculture by identifying cotton pests and diseases using the CFNet-VoV-GCSP-LSKNet-YOLOv8s framework [21]. Although deep learning has been applied to pest recognition, no current method exists specifically for identifying *B. tabaci*.

The Raspberry Pi, developed by the Raspberry Pi Foundation, is a popular embedded microdevice that has enabled students to learn about embedded systems due to its convenience and capabilities. With improved hardware, the Raspberry Pi now supports more complex computing tasks like object detection. Zhu and other researchers have developed the Raspberry Pi-based pest and disease detection and identification system, which recognizes and identifies the pests and diseases of fruits such as longan and litchi [22]. Watawana and Isaksson devised an automated greenish phenotype system for yield estimation, utilizing consumer-grade depth cameras and implementing it on Raspberry Pi devices [23]. Not only was the Raspberry Pi employed as a sensor control center for the construction of an embedded system for corn canopy image acquisition, but it also served as a component of distributed computing and deep learning for monitoring nighttime temperatures in irrigated rice paddies [24, 25].

In this study, we amassed a 1200-image original dataset of cotton whiteflies against cotton leaf backgrounds, each with 1000×1000 resolution, to facilitate accurate identification and quantification. By segmenting each image into 12 slides and employing augmentation techniques like flipping, rotation, and mosaic, we generated around 16,000 images. We further enhanced the You Only Look Once (YOLO) v8 model by incorporating a Swin-Transformer module in the backbone and a P2 structure in the Head. Ablation studies revealed the optimized models superior performance on the whitefly dataset. We also added Slicing Aided Hyper Inference (SAHI) and fine-tuning to detect the small object whitefly. Meanwhile, we randomly selected a few images and conducted an error analysis between manual and machine counting, with a correlation coefficient R^2^ of 0.98. Deploying the algorithm on a Raspberry Pi enabled field-level real-time monitoring. We preliminarily examined whiteflies daily and spatial distribution patterns, conducting fundamental linear regression analysis. This research offers a micro-target insect recognition approach based on the Raspberry Pi, providing valuable insights for cotton whitefly warning monitoring and control, demonstrating the immense potential of deep learning in insect identification.

## Materials and methods

### Plant experimental materials and experimental operation environment

In this study, the cotton variety *Gossypium hirsutum* (TM-1) was cultivated at Zhejiang A&F University (119.72 E, 30.25 N), located in Linan District, Hangzhou City, Zhejiang Province, China. The plants were grown in an open field environment under natural infestation conditions without shade nets. The plants were spaced relatively far apart in pots to minimize insect disturbance when taking photos. The experiment was conducted in July 2023, involving 40 cotton plants. The cotton was planted in May, and 2-month-old plants were used for detection. During the study, a high density of whiteflies was present.

The photos were taken by a Redmi 40 mobile phone, and the image resolution of the dataset is 4000×3000. As for the coding environment, Arch Linux was chosen as an operating system for training and predicting. The CPU was Ryzen 7 4800H, and the GPU was GTX 1650Ti for laptops, whose high precision for float calculation could strengthen the learning ability of the convolution neural network. This programs operations were built on Python 3.9.7, PyTorch 2.0.1, and torchvision 0.15.2. The virtual environment was developed with the Miniconda (23.1.0) in WSL Arch Linux.

The Raspberry Pi 4B was an embedded webcam platform, leveraging an FHD camera to stream real-time images with minimal latency onto a local network. The Raspberry Pi 4B supports 2.4 GHz/5 GHz dual band 802.11ac Wi-Fi, with a maximum wireless bandwidth of 15 MB/s. The raw image stream was broadcasted on a designated port, enabling a local prediction device to continuously analyze the images and display the count of detected pests.

### The whitefly image dataset collecting

**Fig. 1:**
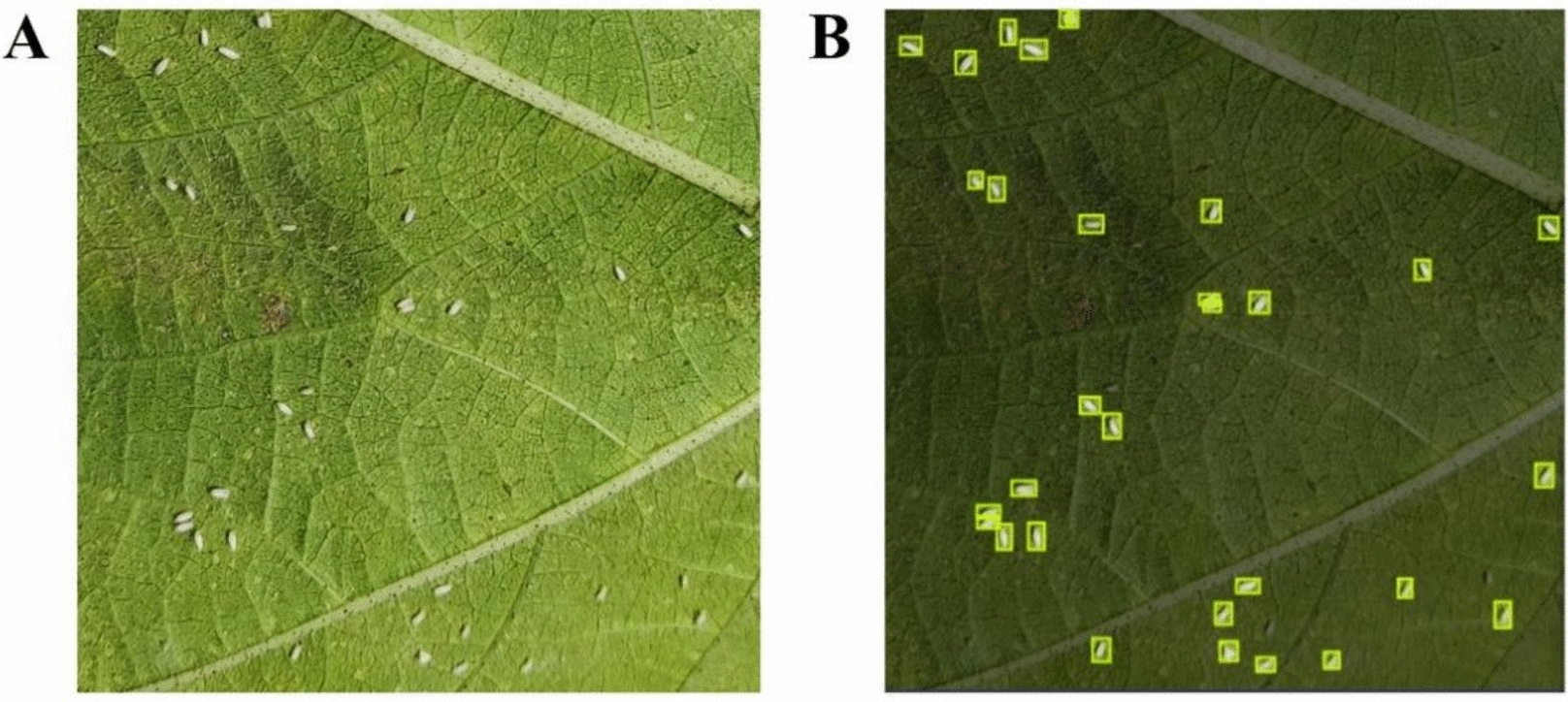
**A** A raw image from the whitefly dataset; **B** an image from the whitefly dataset annotated by Roboflow software

We captured 177 whitefly images chronologically sorted and annotated with specific positions on the cotton plants. For each cotton plant, five images were taken from the top to the bottom and promptly transferred to a laptop. During each capture, the leaves were gently flipped to expose the undersides, requiring cautious handling to prevent whitefly escape. This required a substantial waiting period for the whiteflies to settle and take pictures. Post-capture, the leaves were carefully repositioned to avoid displacement of whiteflies, which might relocate to other leaves, thus resulting in double-counting. A Python script automated the renaming and sorting of these images into separate folders for various applications. The Roboflow tool (https://app.roboflow.com) was applied to label the whitefly dataset (Fig. 1) manually.

### Image preprocessing

**Fig. 2:**
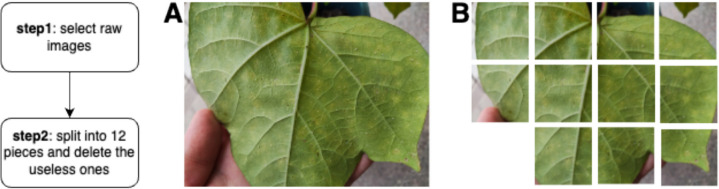
Image preprocessing. **A** A raw image; **B** an image divided into 12 parts

We used the Roboflow software to manually label whitefly images for the whitefly dataset [26]. Each image captured before was split into 12 independent ones, whose size is 1000×1000 pixels, and the useless parts were possibly deleted (Fig. 2). Two thousand one hundred twenty-four images were segmented from the raw data, and 1200 valid images were selected and annotated.

We employed image augmentation techniques to enrich the dataset, aiming to enhance the clarity of whitefly images and improve the models generalization capability. On Roboflow annotation, we utilized computer vision methods such as image flipping, noise addition, and image contrast, brightness, and saturation adjustments for image enhancement. During the initial training phase, we further applied image augmentation techniques, including mosaic, mix-up, random perspective, and HSV augmentation, to increase the diversity of the training dataset and avoid sample imbalance.

### Design and improvements of YOLO v8 model

The model to be present was mainly developed upon YOLO v8, which is applied in object detection and image segmentation [27].

#### Optimizer choice

As for the optimizer, Adam was chosen, which was contained in the YOLO v8 module [28]. Adam is an adaptive algorithm for first-order gradient-based optimization of stochastic objective functions based on adaptive estimates of lower-order moments. Adam reduced training time while increasing the accuracy of the model.

#### Backbone added Swin-Transformer attention module

There are three major parts in the YOLO v8 model: the neck, the Head, and the backbone. The backbone network was aimed at object classification, giving a confidence score for predicting each catalog. The Swin-Transformer attention module was added to the backbone network to detect tiny objects more precisely.

**Fig. 3:**
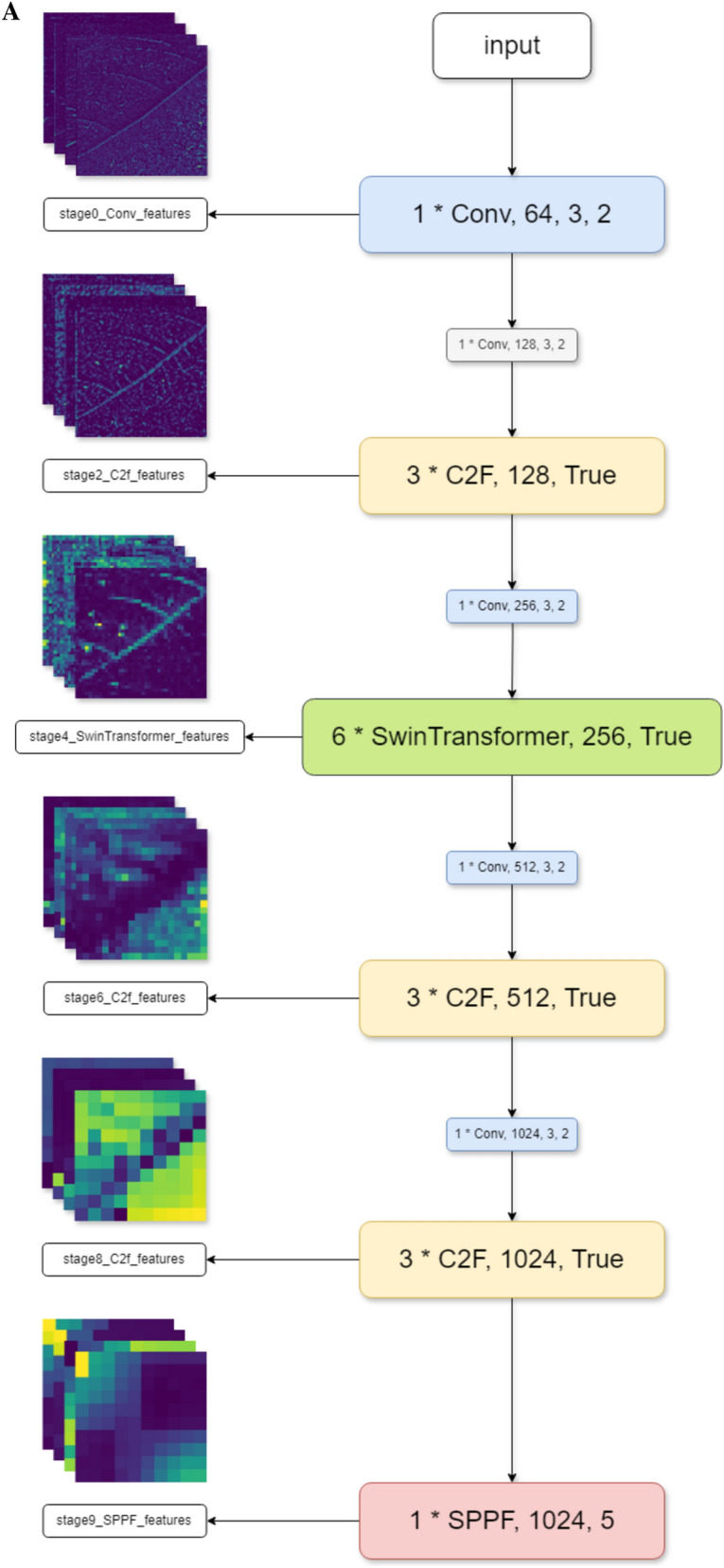

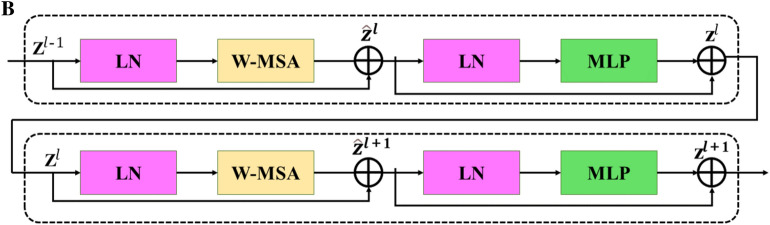
**A** Feature extraction; **B** two successive Swin-Transformer blocks

Transformer, a deep learning architecture proposed in 2017, relies on the multi-head attention mechanism [29]. Lacking recurrent units, the transformer demands less training time than previous recurrent neural networks. Transformer architecture initially performed remarkably well in natural language processing tasks and later proved beneficial t for computer vision tasks, Vision Transformer (ViT) [30]. In 2021, a pure transformer model demonstrated superior performance and greater efficiency than CNNs on machine vision. Swin-Transformer was an implementation offered by Microsoft that addressed the limitations of traditional Transformer implementations, which can be computationally demanding for less efficient GPUs [26]. To deal with those tough situations, Swin-Transformer adopts a shifted window approach to optimize. Thus, combining C2f, CNNs, and Swin-Transformer strengthens the models ability to extract richer features of raw images. Several stages of the feature extraction and two successive Swin-Transformer blocks are displayed below (Fig. 3).

#### Head

**Fig. 4:**
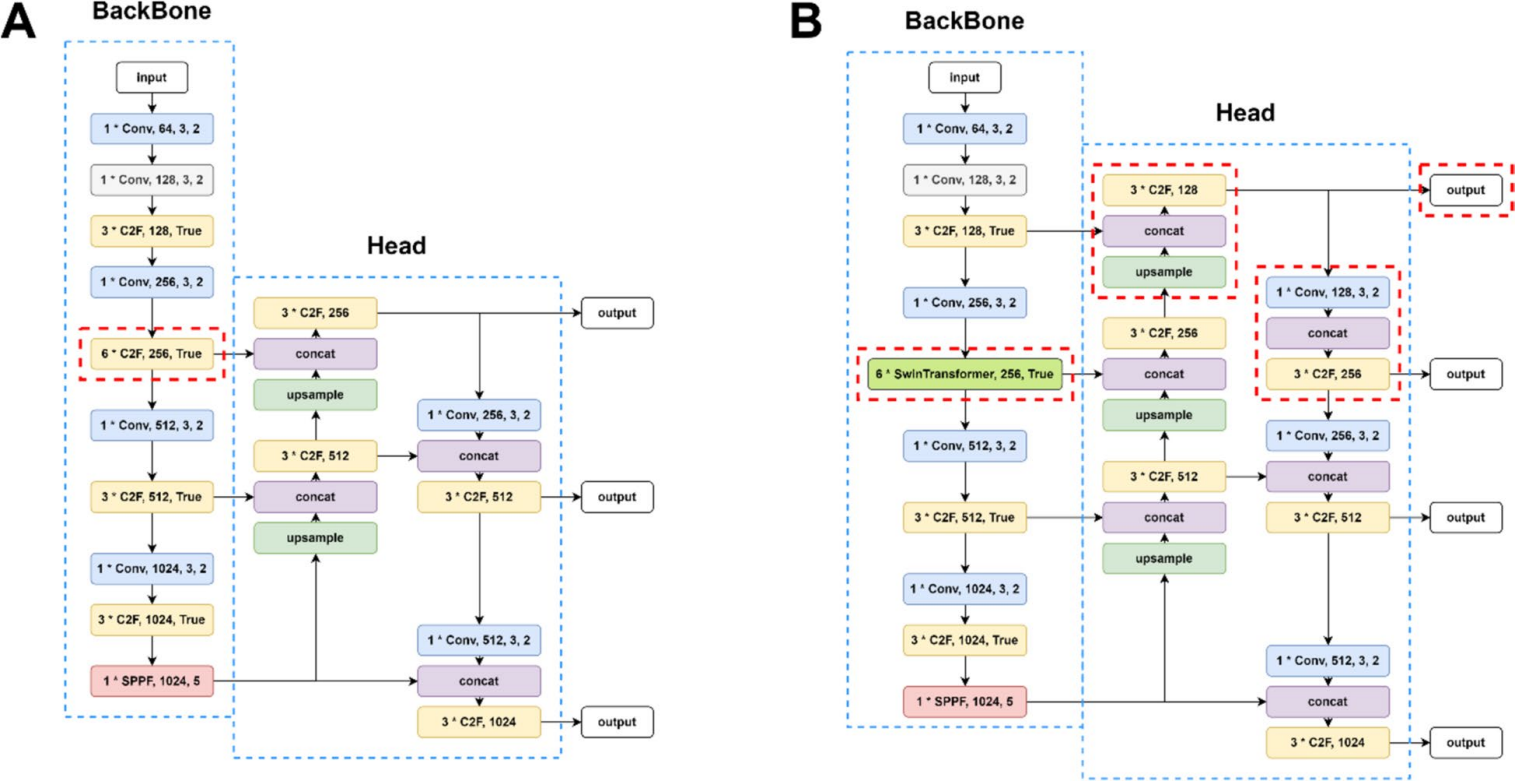
Improvement for the model. **A** The raw model structure of YOLO v8. **B** Improved YOLO v8 model structure

Because our task was to predict tiny objects, another improvement on Head was added. YOLO v8-P2 is a variant of the YOLO v8 model designed for better performance on small objects. Compared to the original YOLO v8 model, YOLO v8P2 includes additional feature pyramid levels, enabling it to detect and localize smaller objects more accurately and better fuse the multi-scale information. The architecture of the improved model is shown below (Fig. 4).

### SAHI applied in whitefly detection

Slice inference technology independently analyzes small regions of an image and fuses the predictions to achieve accurate whole-image predictions. SAHI (Slicing Aided Hyper Inference), an open-source framework, offers a slicing-assisted inference and fine-tuning solution for small object detection [31]. In our study, SAHI was integrated into an enhanced YOLO v8 model to identify small targets precisely. SAHI excels in predicting large-scale static images by optimizing object detection algorithms through slicing, thereby enhancing detection accuracy. Its key functionality is slicing images into manageable regions, running detection on each slice independently, and fusing the outcomes for a comprehensive prediction. SAHIs compatibility and flexibility allow it to seamlessly integrate with models like the YOLO series, optimizing the utilization of computational resources.

### The whitefly detection algorithm deployment in the field facilities

The Raspberry Pi is a credit card-sized computer, a microcomputer motherboard based on ARM, with all the basic functions of a PC. An Real Time Streaming Protocol (RTSP) server is deployed on the Raspberry Pi 4B, continuously capturing video streams through connected camera devices. The camera component is built-in to the Raspberry Pi system, with version number Debian 12 and Kernel 6.6. It transmits them to the local network following the RTSP standard. The video streams are then received by nearby computing devices, running image recognition models to process the video frames immediately. After the model predicts these image frames, they are output to the screen as labeled images with bounding boxes, achieving instant visualization of the prediction results.

To enhance user experience and cross-platform compatibility, a graphical user interface (GUI) software was developed using the PyQt6 module. This software supports running on the Raspberry Pi and works smoothly on multiple operating systems such as Windows and Mac OS. The GUI software is divided into two main display areas: one for displaying image comparisons before and after prediction and the other for printing log information in text form. In addition, the software interface also includes two control buttons, which are used to start and stop the reading and processing of video streams. When the user clicks the start button, the software automatically connects to the Raspberry Pi camera or other available video stream sources and predicts images every 0.5 s. The prediction results will be displayed on the interface in real-time, and related information will also be updated to the text log area simultaneously.

### Model training

We divided the whitefly dataset into a 7:1:2 ratio for training, validation, and testing. We conducted ten independent trainings, each with a different randomly shuffled dataset using five random seeds to achieve higher model performance and enhance its generalization ability. The training epoch parameter was set to 200 epochs. Before training the target model, we employed transfer learning (TL) for pre-training. The core idea of TL is to transfer labeled data or knowledge structures from a related domain to complete or improve the learning effect in the target domain or task. Firstly, we trained the pre-trained model provided by Ultralytics on the COCO dataset and then utilized this pre-trained model to train the target model on the whitefly dataset. We constructed a transformer-based model with randomly initialized parameters but subsequently loaded the corresponding parameters from the pre-trained model. For layers shared between the two models, the parameters remained unchanged and were directly transferred to the new model, while the parameters in unique layers remained randomly initialized. By doing so, we aimed to combine the transformers advantages and the transfer learning efficiency to improve the models performance on the whitefly dataset.

### Analysis methods on manual and machine measurement

Excel and linear regression analysis were used to compare manually counted whiteflies with predictions from the improved deep learning model, generating corresponding scatter plots and linear function graphs. We used linear regression analysis to calculate the difference between manual counting and automatic counting based on the improved YOLO v8s model.

### Whitefly spatial distribution analysis

For the spatial distribution analysis, images were captured from the top (uppermost leaf), middle (three randomly selected leaves from the upper-middle, middle, and lower-middle positions), and bottom (lowermost leaf) parts of each cotton plant. The study included 27 biological replicates, with whitefly counts recorded at 8 AM and 5 PM, as shown in Fig. 9B. In the morning, 55 images from 11 replicates were analyzed, while 40 images from 8 replicates were used in the afternoon. To analyze spatial distribution, average data were calculated, and a one-way ANOVA was performed using IBM SPSS Statistics 27. A students t-test was also conducted using SPSS to compare morning and afternoon counts.

## Results

### The whitefly detection result based on the improved YOLO v8

**Fig. 5:**
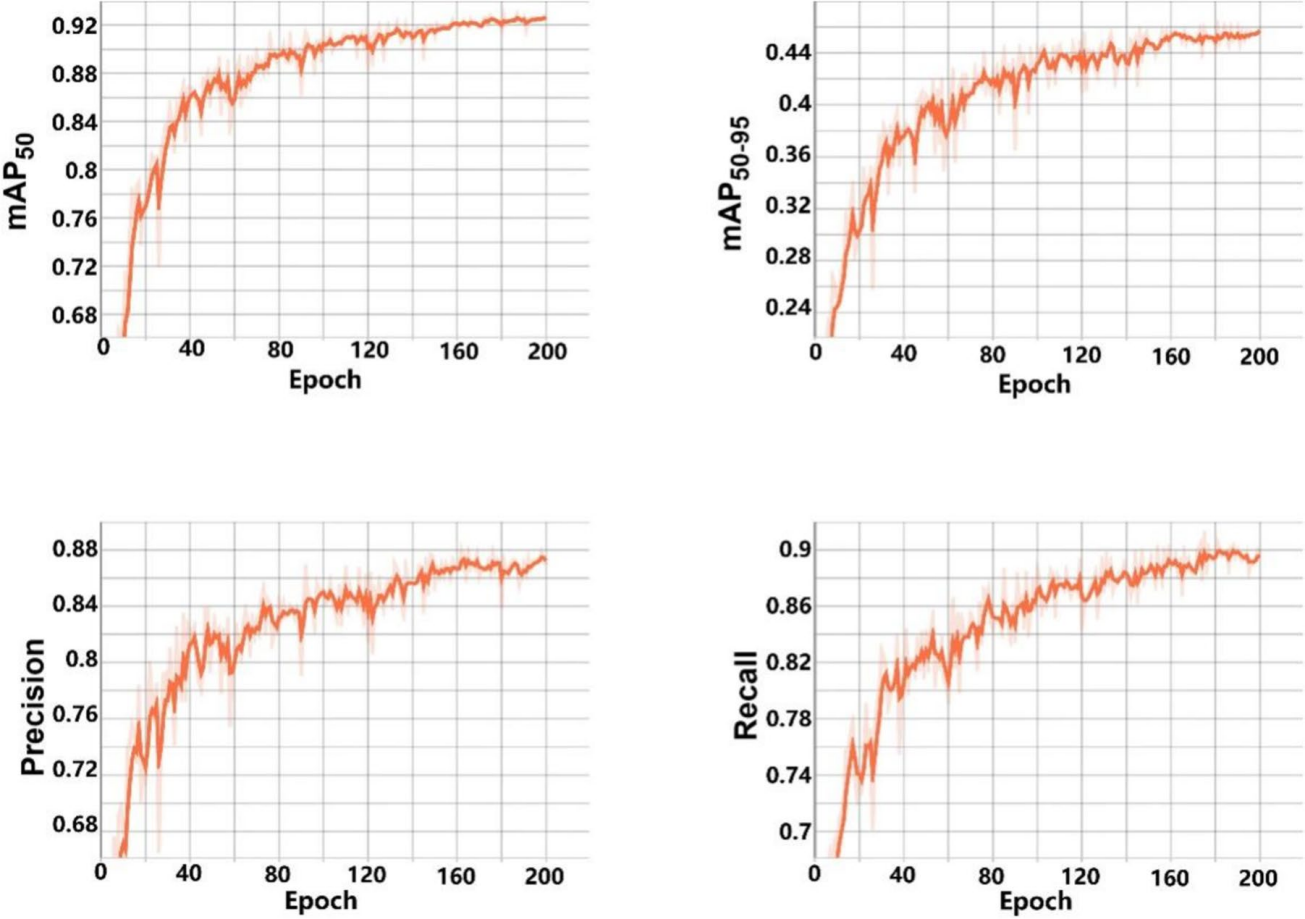
Four evaluation indicators (P, R, mAP_50_, and mAP_50-95_) accompanied by changes in epoch progression

After training 200 epochs, the performance of the improved YOLO v8 was evaluated based on precision (P), recall (R), mAP_50_, and mAP_50-95_. mAP_50_ and mAP_50-95_ are two metrics that can assess the ability and precision of target detection. Figure 5 depicts the training progression of the improved YOLO v8 model. As training epochs accumulate, the metrics of Precision (P), Recall (R), mAP_50_, and mAP_50-95_ typically exhibit a distinct evolution pattern. mAP50 quantifies average accuracy at an IoU threshold of 0.5, while mAP_50-95_ captures average accuracy across IoU thresholds ranging from 0.5 to 0.95. In initial training epochs, such as the first five epochs, due to random weight initialization, P, R, mAP_50_, and mAP_50-95_ may be low, reflecting the models lack of learned features for accurate target identification. As training progresses, these metrics volatility rises, indicating improved target detection performance. Rising P suggests an increase in true positives among predicted positives, while growing R indicates an augmented detection of genuine positive samples. In later epochs, P, R, mAP_50_, and mAP_50-95_ stabilize, indicating the model has attained sufficient knowledge, rendering further metric improvement challenging. After training 200 epochs, the mAP_50_ achieved a value of 0.92, while the mAP_50-95_ attained 0.44. Precision (P) and Recall (R) respectively reached 0.85 and 0.88; the F1 score was 0.86. These results indicate that our model successfully fits the whitefly detection task.

### Ablation experiment results

In our study on deep learning object detection utilizing the whitefly dataset, we initially conducted baseline experiments based on YOLO v8n and YOLO v8s models. The outcomes demonstrate that YOLO v8n achieved commendable performance on critical metrics such as Precision (0.83), Recall (0.77), mAP_50_ (0.82), mAP_50-95_ (0.38), and F1-score (0.8). We further augmented the number of layers to enhance the model and adopted the YOLO v8s variant. This refinement significantly bolstered recall, mAP_50_, mAP_50-95_, and F1-score, while precision remained relatively stable. Through ablation experiments, it was verified that these structural improvements not only increased the complexity of the model but also significantly improved all five evaluation indicators.

Surprisingly, when enhancing the YOLO v8s model, experiments that only introduced the Swin-Transformer module showed a decrease in all evaluation metrics compared to the YOLO v8n version containing the Swin-Transformer and the original YOLO v8s baseline.

Specifically, in the YOLO v8s model that integrates Swin-Transformer and P2 structure, precision increased to a maximum value of 0.87, recall reached 0.89, and mAP_50_ and mAP_50-95_ increased to 0.92 and 0.45, respectively. At the same time, the F1-score also reached the highest of 0.88. These improved indicators demonstrate that the YOLO v8 model, which has been structurally optimized, performs well in detecting small target objects such as white planthoppers and has high recognition accuracy (Table 1).

**Table 1:** The detection results of different models in the ablation experiment

Baseline	Improved structure	P	R	mAP50	mAP50-95	F1	Parameters
YOLO v8n	–	0.83	0.77	0.82	0.38	0.8	3,011,043
YOLO v8s		0.83	0.79	0.84	0.41	0.81	11,135,987
YOLO v8n	Swin-Transformer	0.82	0.74	0.79	0.36	0.78	3,004,133
YOLO v8s		0.62	0.69	0.68	0.29	0.67	11,105,783
YOLO v8n	P2	0.81	0.80	0.83	0.38	0.81	2,926,692
YOLO v8s		0.85	0.88	0.92	0.44	0.86	10,637,236
YOLO v8n	Swin-Transformer+P2	0.82	0.82	0.85	0.40	0.83	2,919,782
YOLO v8s		0.87	0.89	0.92	0.45	0.88	10,607,032

### Manual vs. machine counting results

**Fig. 6:**
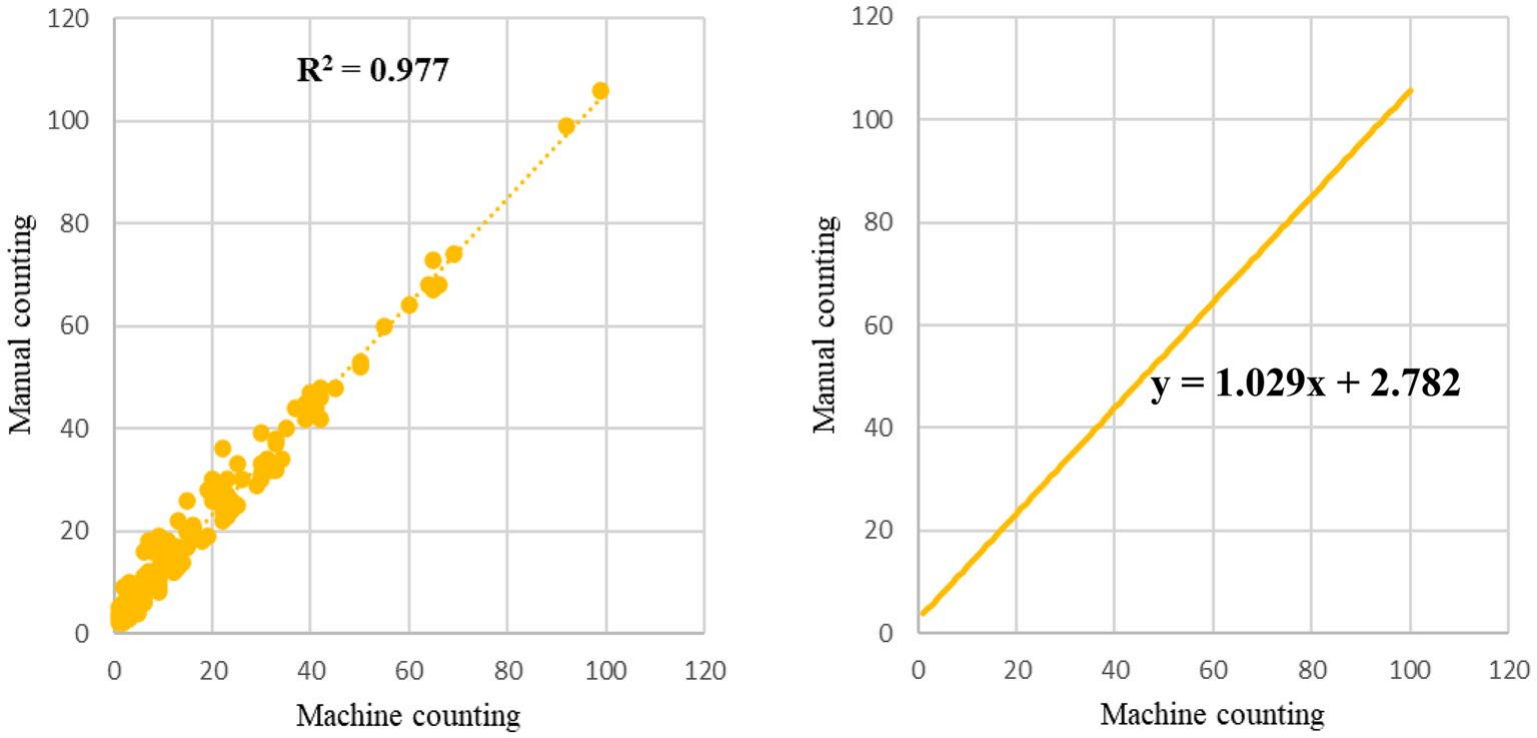
Comparison between manual and machine counting of the whitefly dataset obtained in regression function image

The results show that deep learning measurement is high-speed in terms of the measurement cycle, while manual counting consumes a lot of time and labor (Fig. 6). We discovered that the linear regression function was y=1.029x+2.782, and the square of the correlation coefficient was 0.977. As a result, no substantial difference was observed in the error between manual and deep learning vision measurements (Fig. 6).

### Real-time detection of whiteflies using the Raspberry Pi

**Fig. 7:**
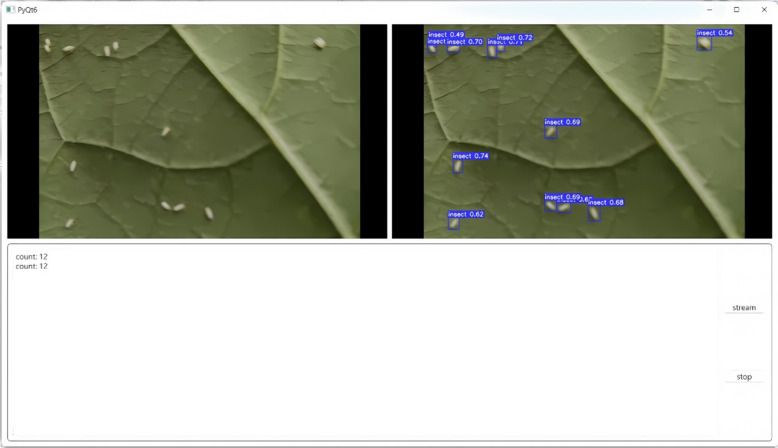
The detection result based on GUI

**Fig. 8:**
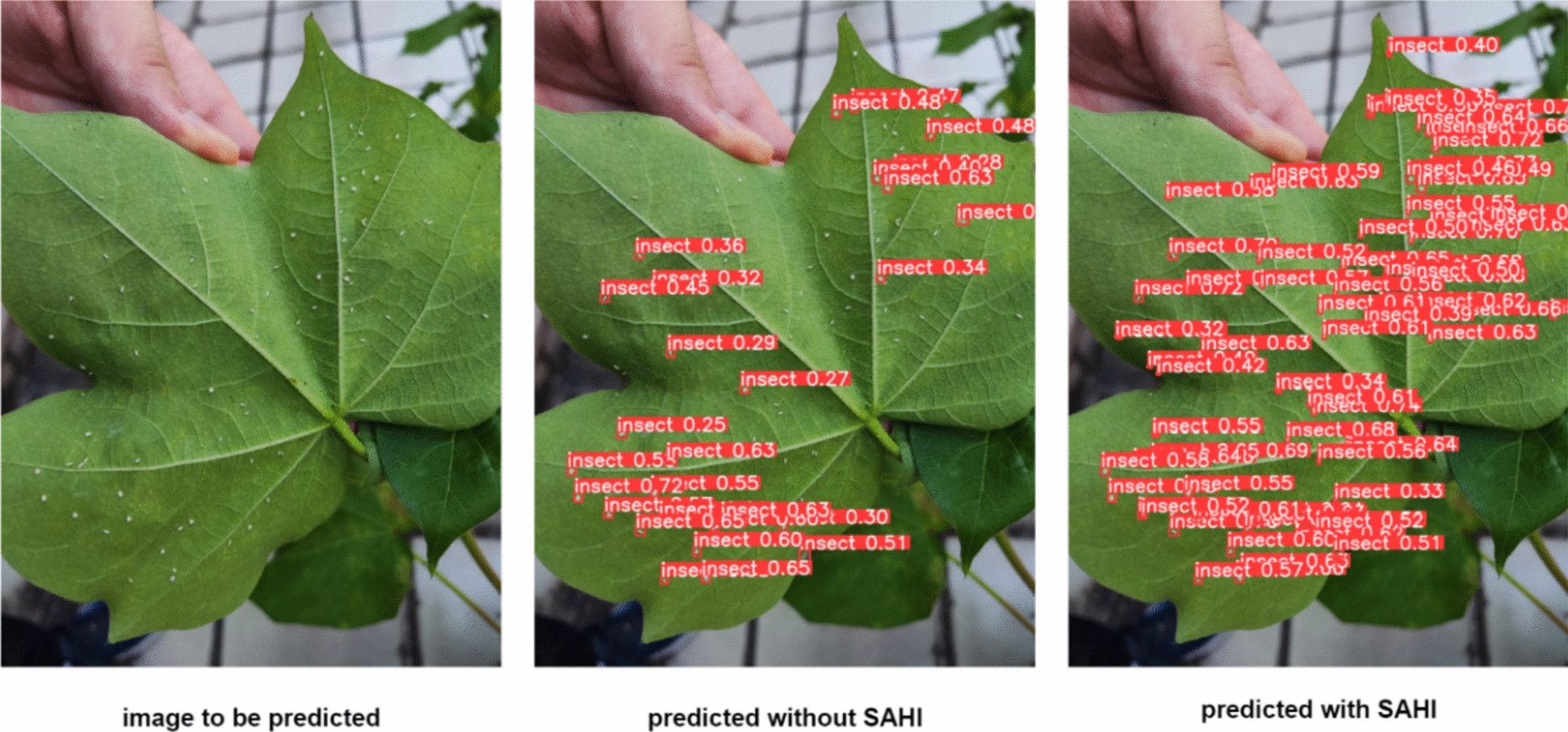
A comparison between the predicted results of SAHI exists

Our study successfully resulted in a GUI-based system employing a camera module connected to a Raspberry Pi, which continuously captures video footage of cotton leaves (Fig. 7). By processing this footage with streaming visualization, the system effectively detects whiteflies on the leaves. The integration of the enhanced YOLO v8s detection algorithm with the SAHI small object detection algorithm leverages advanced deep learning techniques, enabling accurate identification and localization of whiteflies within the captured frames (Fig. 8). Detected whiteflies are highlighted with bounding boxes on the video feed, making it easy for users to spot infestations. Additionally, we observed that detection results are synchronous, with accuracy heavily dependent on the distance between the camera and the target cotton leaf. The optimal distance between the camera and the target leaf is 17 cm, whose error is 0.05. When the distance is less than 12 cm, the error rate increases to 0.79. Conversely, when the distance exceeds 20 cm, the error rate also reaches 0.41.

### Attempts to study whitefly distribution on cotton

**Fig. 9:**
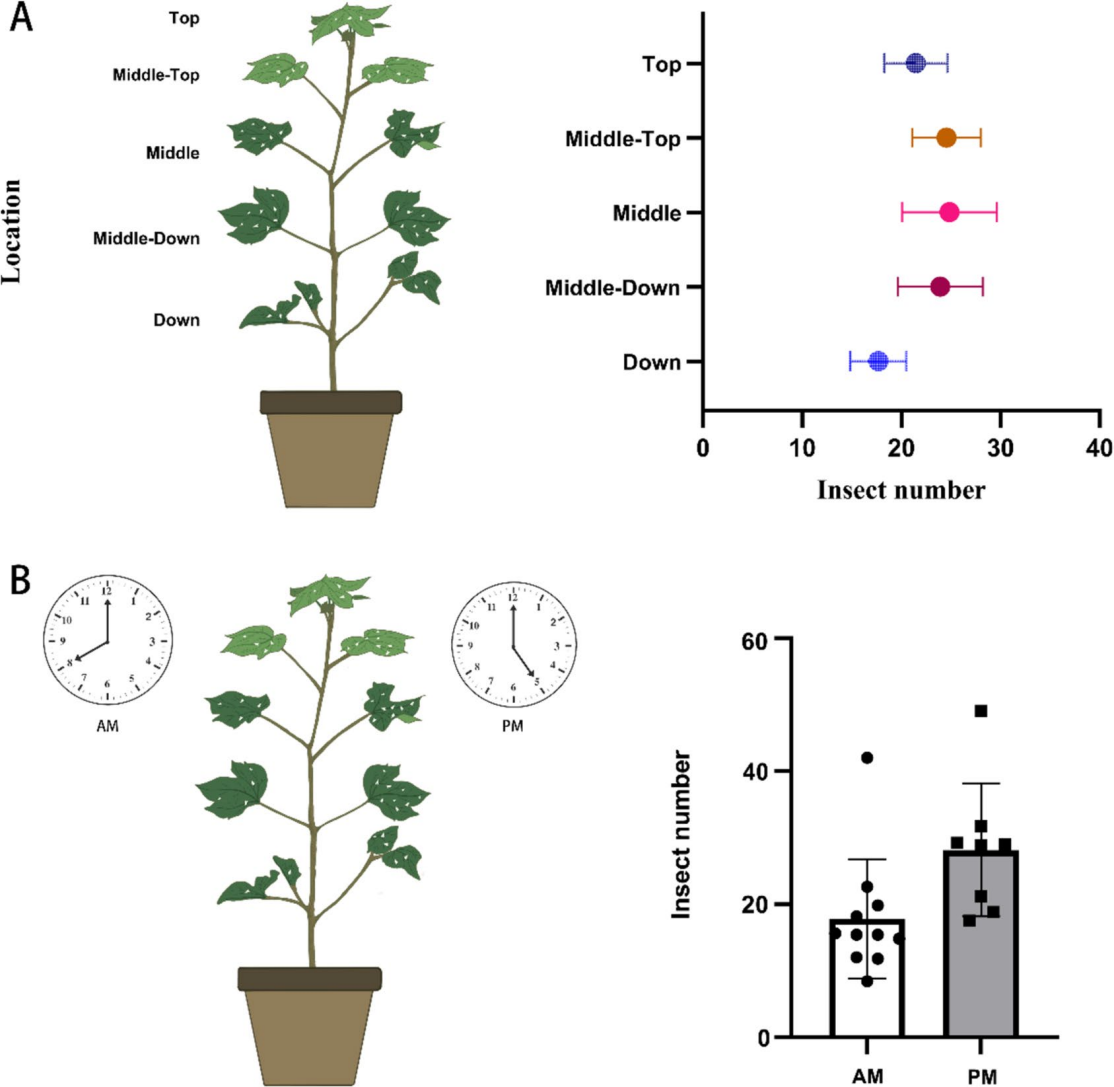
Initial analysis of whitefly distribution across different times and locations, with insect numbers representing whiteflies per leaf. Location: different area of the cotton plants from the top to bottom. AM: 8 in the morning. PM: 5 in the afternoon

We conducted a small-scale preliminary test to evaluate the whitefly detection models practical applicability. Twenty-seven cotton plants and corresponding images were selected to observe pest distribution on different parts of the cotton leaves. After processing the images for pest detection, we analyzed the whitefly counts statistically. The results indicated that the cotton plants average number of whiteflies on the top, upper-middle, middle, lower-middle, and bottom leaves were 21.44, 24.56, 24.85, 23.93, and 17.67, respectively. These findings suggest that whiteflies are relatively more concentrated in the upper-middle, middle, and lower-middle regions, though the differences between the groups were not statistically significant (p=0.638). Additionally, we compared the whitefly counts at 8 AM and 5 PM, with average numbers of 19.51 and 28.15, respectively. Statistical analysis indicates that the number of pests on the leaves in the afternoon is significantly greater than in the morning (p=0.03) (Fig. 9).

## Discussion

Previous studies have made significant progress in the automatic identification of pests. For example, Pattnaik et al. developed a transfer learning framework using a pre-trained deep CNN to classify pests in tomato plants [32], while Tetila et al. utilized a deep learning model to detect and classify five types of soybean pests using UAV images [33]. Similarly, Badgujar et al. identified common stored product insects through automated deep learning methods [34]. Despite these advancements, reliable monitoring systems for cotton whiteflies have lagged. This study offers a cost-effective, flexible, and scalable solution for detecting cotton whiteflies, facilitating real-time monitoring, remote control, and data-driven decision-making with minimal power consumption.

In this project, we selected the state-of-the-art YOLO v8 deep learning model to ensure both the speed and accuracy of whitefly detection. Considering that the model will eventually be deployed on low-cost, resource-constrained devices such as the Raspberry Pi, we optimized the YOLO v8 model accordingly. We utilized PyTorchs Automatic Mixed Precision (AMP) mechanism, which allows efficient operation even on older GPUs like the GTX 1650Ti. Additionally, we identified the need for a strategy to optimize the training and inference processes further to accelerate research progress. Considering the small size of the dataset, we only used slight data augmentation methods (such as flipping and rotating) with the aim of expanding the dataset. Appropriate data augmentation enhances the dataset and reduces overfitting likelihood, while the baseline includes regularization to prevent overfitting. The good evaluation performance result indicates no overfitting occurred after data augmentation. However, our analysis reveals that the Swin-Transformer, despite its strong global feature extraction and data retention capabilities, tends to overfit small datasets due to its numerous parameters and focus on global features. This also explains the decline in Precision (P) and Recall (R) after integrating Swin-Transformer into the backbone. This makes it less suitable for small object detection tasks. To address this issue, we introduced a P2 structure in the Head of YOLO v8s, and our results indicate that this modification contributes to improved evaluation metrics, including precision, mAP_50_, and F1-score. Furthermore, integrating the SAHI algorithm was pivotal in refining the detection of small objects, which was particularly important for effective deployment on resource-constrained devices like the Raspberry Pi. The high correlation between manual and machine counting (R^2^=0.977) confirms the reliability of our automated detection approach, suggesting its strong potential for practical use in agricultural settings.

We then conducted a preliminary study of whitefly distribution, finding a concentration of whiteflies in the cotton plants middle-top, middle, and middle-down sections during the afternoon. Although no significant differences were observed between spatial comparison groups, our results suggest this method has strong potential for future field research. The observed temporal and spatial distribution patterns offer valuable insights for targeted pest management strategies, which could optimize control measures and enhance crop protection. However, the studys limitations, including the need for larger datasets and the challenge of maintaining accuracy under varied conditions, highlight areas for further research. Future work should focus on collecting more extensive samples across different times and plant parts to understand whitefly distribution patterns better. Additionally, applying this method to various cotton varieties could help assess host-plant resistance. Similarly, this study can also be extended to other plants infected with whiteflies, such as tobacco, cucumber, tomato, eggplant, and melon.

## Conclusion

This study developed a method for detecting and counting cotton whiteflies by augmenting the dataset and refining the YOLO v8s model, significantly enhancing detection capabilities. The high correlation between manual and automated counts demonstrates the accuracy of this approach. The deep learning model was successfully deployed on a Raspberry Pi, paving the way for future applications in compact, cost-effective instruments used in the field. Building on this, the method also attempts to analyze the distribution patterns of cotton whiteflies, aiming to provide valuable insights and solutions for targeted pest management.

## Data Availability

No datasets were generated or analysed during the current study.

## References

[1] Balls WL. The development and properties of the cotton fibre. Nature. 1914;93(2325):3089.

[2] Feng Y, Wang Y, Lu H, Li J, Akhter D, Liu F, Zhao T, Shen X, Li X, Whelan J, et al. Assembly and phylogenomic analysis of cotton mitochondrial genomes provide insights into the history of cotton evolution. Crop J. 2023;11(6):178292.

[3] Dinsdale A, Cook L, Riginos C, Buckley YM, Barro PD. Refined global analysis of *Bemisia tabaci* (Hemiptera: Sternorrhyncha: Aleyrodoidea: Aleyrodidae) mitochondrial cytochrome oxidase 1 to identify species level genetic boundaries. Ann Entomol Soc Am. 2010;103(2):196208.

[4] Gong C, Guo Z, Hu Y, Yang Z, Xia J, Yang X, Xie W, Wang S, Wu Q, Ye W, et al. A Horizontally transferred plant fatty acid desaturase gene steers whitefly reproduction. Adv Sci. 2024;11(10):2306653. 10.1002/advs.202306653 PMC10933598 38145364

[5] Nakatumba-Nabende J, Tusubira JF, Babirye C, Nsumba S, Abu C. O. A dataset of cassava whitefly count images. Data Br. 2022;41:107911. 10.1016/j.dib.2022.107911. 10.1016/j.dib.2022.107911 PMC8845108 35198687

[6] Chu D, Zhang YJ, Brown JK, Cong B, Xu B-Y, Wu Q-J, Zhu GR. The introduction of the exotic Q biotype of *Bemisia tabaci* from the Mediterranean region into china on ornamental crops. Fla Entomol. 2006;89(2):16874.

[7] Li J, Zhu L, Hull JJ, Liang S, Daniell H, Jin S, Zhang X. Transcriptome analysis reveals a comprehensive insect resistance response mechanism in cotton to infestation by the phloem feeding insect *Bemisia tabaci* (whitefly). Plant Biotechnol J. 2016;14(10):195675. 26923339 10.1111/pbi.12554 PMC5042180

[8] Shukla AK, Upadhyay SK, Mishra M, Saurabh S, Singh R, Singh H, Thakur N, Rai P, Pandey P, Hans AL, et al. Expression of an insecticidal fern protein in cotton protects against whitefly. Nat Biotechnol. 2016;34(10):104651. 27598229 10.1038/nbt.3665

[9] Kumar A, Birah A, Tanwar RK, Khokhar MK, Singh S, Monga D, Kumar R, Arora JK. Validation of IPM strategy in Bt cotton in whitefly (*Bemisia tabaci*) hot spot of North-West India. Indian J Agric Sci. 2021;91(7):108892.

[10] Guo L, Lv H, Tan D, Liang N, Guo C, Chu D. Resistance to insecticides in the field and baseline susceptibility to cyclaniliprole of whitefly *Bemisia tabaci* (Gennadius) in China. Crop Prot. 2020;130: 105065.

[11] Grover S, Jindal V, Banta G, Taning CNT, Smagghe G, Christiaens O. Potential of RNA interference in the study and management of the whitefly, *Bemisia tabaci.* Arch Insect Biochem Physiol. 2019;100(2):e21522. 10.1002/arch.21522. 10.1002/arch.21522 30484903

[12] Li D, Li HY, Zhang JR, Wu YJ, Zhao SX, Liu SS, Pan LL. Plant resistance against whitefly and its engineering. Front Plant Sci. 2023;14:1232735. 10.3389/fpls.2023.1232735. 10.3389/fpls.2023.1232735 PMC10498545 37711302

[13] Mahmood MA, Awan MJA, Mansoor S. BioClay: next-generation crop protection strategy. Trends Plant Sci. 2022;27(11):10902. 35981953 10.1016/j.tplants.2022.08.003

[14] Zhang X-M, Lövei GL, Ferrante M, Yang N-W, Wan F-H. The potential of trap and barrier cropping to decrease densities of the whitefly *Bemisia tabaci* MED on cotton in China. Pest Manag Sci. 2020;76(1):36674. 31219649 10.1002/ps.5524

[15] Wang N, Cao H, Huang X, Ding M. Rapeseed flower counting method based on GhP2-YOLO and StrongSORT algorithm. Plants. 2024;13:2388. 39273871 10.3390/plants13172388 PMC11396797

[16] Wang N, Liu H, Li Y, Zhou W, Ding M. Segmentation and phenotype calculation of rapeseed pods based on YOLO v8 and mask R-convolution neural networks. Plants. 2023;12(18):3328. 37765490 10.3390/plants12183328 PMC10537308

[17] Shen Y, Zhou H, Li J, Jian F, Jayas DS. Detection of stored-grain insects using deep learning. Comput Electron Agric. 2018;145:31925.

[18] Amarathunga DC, Ratnayake MN, Grundy J, Dorin A. Fine-grained image classification of microscopic insect pest species: western flower thrips and plague thrips. Comput Electron Agric. 2022;203: 107462.

[19] Sun L, Cai Z, Liang K, Wang Y, Zeng W, Yan X. An intelligent system for high-density small target pest identification and infestation level determination based on an improved YOLOv5 model. Expert Syst Appl. 2024;239: 122190.

[20] Devi MG, Rustia DJA, Braat L, Swinkels K, Espinosa FF, van Marrewijk BM, Hemming J, Caarls L. Eggsplorer: a rapid plantinsect resistance determination tool using an automated whitefly egg quantification algorithm. Plant Methods. 2023;19(1):49. 37210517 10.1186/s13007-023-01027-9 PMC10200050

[21] Li R, He Y, Li Y, Qin W, Abbas A, Ji R, Li S, Wu Y, Sun X, Yang J. Identification of cotton pest and disease based on CFNet-VoV-GCSP-LSKNet-YOLOv8s: a new era of precision agriculture. Front Plant Sci. 2024;15:1348402. 38444536 10.3389/fpls.2024.1348402 PMC10913016

[22] Zhu D, Xie L, Chen B, Tan J, Deng R, Zheng Y, Hu Q, Mustafa R, Chen W, Yi S, et al. Knowledge graph and deep learning based pest detection and identification system for fruit quality. Internet Things. 2023;21: 100649.

[23] Watawana B, Isaksson M. Automated microgreen phenotyping for yield estimation using a consumer-grade depth camera. Smart Agric Technol. 2024;7: 100384.

[24] Quiñones C, Adviento-Borbe MA, Larazo W, Harris RS, Mendez K, Cunningham SS, Campbell ZC, Medina-Jimenez K, Hein NT, Wagner D, et al. Field-based infrastructure and cyberphysical system for the study of high night air temperature stress in irrigated rice. Plant Phenome J. 2023;6(1): e20085.

[25] Tausen M, Clausen M, Moeskjær S, Shihavuddin ASM, Dahl AB, Janss L, Andersen SU. Greenotyper: image-based plant phenotyping using distributed computing and deep learning. Front Plant Sci. 2020;11:1181. 32849731 10.3389/fpls.2020.01181 PMC7427585

[26] Alexandrova S, Tatlock Z, Cakmak M. RoboFlow: a flow-based visual programming language for mobile manipulation tasks. In: 2015 IEEE international conference on robotics and automation (ICRA): 2630 May 2015; 2015. p. 553744.

[27] Zhou Y, Zhu W, He Y, Li Y. YOLOv8-based spatial target part recognition. In: 2023 IEEE 3rd international conference on information technology, big data and artificial intelligence (ICIBA): 2628 May 2023; 2023. p. 16847.

[28] Kingma DP, Ba JJC: Adam: a method for stochastic optimization. 2014. arXiv:1412.6980.

[29] Vaswani A, Shazeer NM, Parmar N, Uszkoreit J, Jones L, Gomez AN, Kaiser L, Polosukhin I. Attention is all you need. In: Neural information processing systems; 2017.

[30] Dosovitskiy A, Beyer L, Kolesnikov A, Weissenborn D, Zhai X, Unterthiner T, Dehghani M, Minderer M, Heigold G, Gelly S, et al. An image is worth 16×16 words: transformers for image recognition at scale. 2020. arXiv:2010.11929.

[31] Akyon FC, Altinuc SO, Temizel A. Slicing aided hyper inference and fine-tuning for small object detection. In: 2022 IEEE international conference on image processing (ICIP): 1619 Oct. 2022; 2022. p. 96670.

[32] Pattnaik G, Shrivastava VK, Parvathi K. Transfer learning-based framework for classification of pest in tomato plants. Appl Artif Intell. 2020;34(13):98193.

[33] Tetila EC, Machado BB, Astolfi G, Belete NADS, Amorim WP, Roel AR, Pistori H. Detection and classification of soybean pests using deep learning with UAV images. Comput Electron Agric. 2020;179: 105836.

[34] Badgujar CM, Armstrong PR, Gerken AR, Pordesimo LO, Campbell JF. Identifying common stored product insects using automated deep learning methods. J Stored Prod Res. 2023;103: 102166.

